# Comparing estimates of child mortality reduction modelled in LiST with pregnancy history survey data for a community-based NGO project in Mozambique

**DOI:** 10.1186/1471-2458-11-S3-S35

**Published:** 2011-04-13

**Authors:** Jim Ricca, Debra Prosnitz, Henry Perry, Anbrasi Edward, Melanie Morrow, Pieter Ernst, Leo Ryan

**Affiliations:** 1 ICF Macro, 11785 Beltsville Drive, Calverton, MD 20705, USA; 2 Johns Hopkins Bloomberg School of Public Health, Department of International Health, Baltimore, MD 21205, USA; 3 World Relief, 7 E. Baltimore St., Baltimore MD 21202, USA; 4 World Relief, Chokwe, Gaza Province, Mozambique

## Abstract

**Background:**

There is a growing body of evidence that integrated packages of community-based interventions, a form of programming often implemented by NGOs, can have substantial child mortality impact. More countries may be able to meet Millennium Development Goal (MDG) 4 targets by leveraging such programming. Analysis of the mortality effect of this type of programming is hampered by the cost and complexity of direct mortality measurement. The Lives Saved Tool (LiST) produces an estimate of mortality reduction by modelling the mortality effect of changes in population coverage of individual child health interventions. However, few studies to date have compared the LiST estimates of mortality reduction with those produced by direct measurement.

**Methods:**

Using results of a recent review of evidence for community-based child health programming, a search was conducted for NGO child health projects implementing community-based interventions that had independently verified child mortality reduction estimates, as well as population coverage data for modelling in LiST. One child survival project fit inclusion criteria. Subsequent searches of the USAID Development Experience Clearinghouse and Child Survival Grants databases and interviews of staff from NGOs identified no additional projects. Eight coverage indicators, covering all the project’s technical interventions were modelled in LiST, along with indicator values for most other non-project interventions in LiST, mainly from DHS data from 1997 and 2003.

**Results:**

The project studied was implemented by World Relief from 1999 to 2003 in Gaza Province, Mozambique. An independent evaluation collecting pregnancy history data estimated that under-five mortality declined 37% and infant mortality 48%. Using project-collected coverage data, LiST produced estimates of 39% and 34% decline, respectively.

**Conclusions:**

LiST gives reasonably accurate estimates of infant and child mortality decline in an area where a package of community-based interventions was implemented. This and other validation exercises support use of LiST as an aid for program planning to tailor packages of community-based interventions to the epidemiological context and for project evaluation. Such targeted planning and assessments will be useful to accelerate progress in reaching MDG4 targets.

## Background

Although there are encouraging trends in some key countries, meeting Millennium Development Goal (MDG) 4 for reduction of child mortality will be challenging, given current trends.[1] Community-based intervention packages are not commonly implemented at large scale, although recent evidence demonstrates that they are effective for neonatal and child mortality reduction at moderate scale in various resource-constrained settings. [2,3] This has prompted calls for greater emphasis on community-level delivery, especially preventive interventions and integrated strategies. [2,4,5]

Analysis of effectiveness of this type of programming is hampered by its cost and complexity. It is difficult to estimate the mortality impact of packages of interventions in realistic field settings, as well as effectiveness of component interventions within packages. [6] Projects implementing interventions under these conditions usually lack the resources necessary to carry out mortality impact evaluations. The Lives Saved Tool (LiST) produces mortality reduction estimates by modelling the mortality effect of increases in population coverage for key child health interventions. LiST calculates this by combining coverage change data with data on effectiveness of each intervention against common serious child illnesses, and country-specific cause of death profiles. This is explained in detail elsewhere. [7] By producing intuitive and equivalent outputs from otherwise disparate data, such as the percentage reduction in mortality rates and number of deaths averted, LiST facilitates comparisons that are otherwise difficult to make. Population based surveys in which mortality is directly measured are costly, difficult, and time-consuming, and LiST modelling could be an attractive alternative to estimate mortality reduction.

In order to validate LiST-produced estimates of child mortality reduction in community-based NGO programming, a search was done of such projects with complete coverage data for their child health interventions and independent child mortality reduction estimates. One met criteria for inclusion.

## Methods

### Search for community-based NGO projects

Projects with data available for validation of LiST were sought based on the following criteria: 1. Study was of a community-based NGO child health project; 2. Baseline and endline population coverage indicators were available for at least two child health interventions; 3. Mortality data were available at least at baseline and endline and independently verified. A comprehensive search of the published literature had been run on PubMed by one of the authors (HP) for effectiveness of community-based interventions. The 3,000 articles from this search were reviewed and one project was identified that fit selection criteria, a USAID-funded child survival project implemented by World Relief in Mozambique from 1999-2003.[8] A search for similar projects not published in the peer-reviewed literature was then run on USAID’s Development Experience Clearinghouse database (http://dec.usaid.gov) and Child Survival and Health Grants database (http://www.mchipngo.net). Five additional candidate projects were identified. Project documentation was reviewed and knowledgeable staff interviewed. None of these additional projects met inclusion criteria. Table 1 shows selected key characteristics of this project which was not a research project and had no control or comparison group. The project intervened on all major causes of under-five mortality in the area (neonatal conditions, malaria, pneumonia, diarrhea, and measles) except HIV/AIDS. Direct mortality data were available from an independent retrospective mortality assessment carried out in 2004 by a research team from the Mozambican National Institute of Statistics, Ministry of Health, World Relief and other NGOs, and designed by collaborators from Johns Hopkins School of Public Health. This research team used a pregnancy history survey adapted from the birth history in the women’s questionnaire of the 2003 Mozambique Demographic and Health Survey.

**Table 1:** Key characteristics, strategies, interventions, and results of World Relief Mozambique Vurhonga II project (explained in detail in Edward, et. al.[7])

**Location**
Community-based maternal child health project covering all 48 villages of Chokwe District (excluding Chokwe town), Gaza Province, Mozambique

**Key Dates**
• Funding from October 1999 – September 2003
• After initial planning and baseline studies, project implementation began March 2000
• Population surveys for coverage of key maternal child health services and behaviors in October 1999 (baseline) and July 2003 (endline)
• Additional evaluation studies conducted in May 2004: Retrospective complete pregnancy history survey, mortality results analyzed from March 1998 to February 2004, and reported in six separate 12 month periods
**Main Project Strategies**
• Health related behavior change of mothers of children under five through 173 Care Groups (mothers’ groups with 10-15 volunteers each) trained in monthly supervisory visits, whose members performed monthly visits to 8-10 households in immediate vicinity
• Train health workers and religious leaders in health counseling techniques and content
• Outreach and community-facility links through training of *socorristas* (community outreach workers) in health posts and formation of village health committees
• Strengthen first level of facility-based health care through establishment of health posts in villages that lacked them and health worker training in IMCI
• Train traditional birth attendants and build small delivery rooms with cement floors in several villages for use by project-trained TBAs
**Technical Interventions**
• Nutrition promotion and community-based nutritional rehabilitation
• Promotion of improved care seeking for sick children
• Immunization
• AIDS prevention messaging
• Latrine construction
• TBAs: clean deliveries and essential obstetric and neonatal care (clean cord care, drying and wrapping newborn, skin-to-skin contact, immediate breastfeeding)
• Community case management of diarrhea and pneumonia
• Care of children with diarrhea: promotion of ORT and nutritional support
**Selected Key Results/Outputs**
• Monthly home visits by Care Group (mothers’ group) members, with 100% coverage of households with children under five throughout project period
• Village health committee coverage increased from 0 to 95%
• Outreach workers (*socorristas*) increased in number from 3 to 32
• Increase in access to trained providers of care for sick children from 65% to 99%
• Health providers trained in IMCI increased from 0% to 100% in project area
**Main health activities of other organizations in Gaza District during project period**
• Oxfam assisted in distribution of ITNs to all women of fertile age and children under 5.
• NGO assistance to MOH – train *socorristas* in community-based child health activities.
• National vaccination campaigns, polio eradication campaigns x 2

### Coverage data used for modeling in LiST

The project collected population coverage data on seven LiST interventions pertaining to its areas of intervention. Coverage for an eighth LiST intervention (education for complementary child feeding) was not available, but data collected for increased food intake during previous pregnancy, another nutrition education intervention included in its nutrition education package (Table 2), was used as a proxy. These eight indicators cover all project technical intervention areas.

**Table 2:** World Relief Mozambique Vurhonga II project coverage data and mapping to LiST indicators for modelling

Project coverage indicator	LiST indicator	2000, % (95% CI)	2003, % (95% CI)
Children with diarrhea treated with ORT	Children with diarrhea treated with ORT or Recommended Home Fluids	53 (44 – 62)	94 (90 – 98)
Households with latrine	Improved excreta disposal (latrine/toilet)	28 (10 – 46)	75 (70 – 80)
Households with children that own an insecticide treated net	Households with children that own an insecticide treated net	0 (-)	80 (65 – 85)
Children with fever treated at health facility within 24 hours	Children with fever treated with antimalarial	38 (25 – 51)	95 (86 – 100)
Children with fast or difficult breathing treated at health facility within 24 hours	Antibiotics for pneumonia	26 (14 – 38)	60 (35 – 85)
Delivery of last child by trained birth attendant (trained in clean delivery, immediate breastfeeding, thermal care)	Clean home delivery (LiST also includes delivery of immediate breastfeeding and thermal care in this indicator)	65 (60 – 70)	87 (83 – 91)
Children fully immunized according to national EPI scheme	Measles coverage	74 (66 – 82)	89 (84 – 94)
Mothers reporting increased food intake in last pregnancy	Coverage of this indicator was entered in LiST as complementary feeding since the project’s nutrition education interventions included nutrition in pregnancy and complementary feeding	44 (38 – 50)	82 (78 – 86)

Data sources: Edward, et. al. [7] Indicator values for children treated for fever, treatment for pneumonia, and ownership of insecticide treated net were re-analyzed to fit LiST definitions more closely. KPC survey done in October 1999 and project activities started in March 2000, so baseline values for LiST modelling were dated to 2000. Final values from KPC done in July 2003. 95% CIs calculated using the Z statistic.

Coverage data was collected at baseline and endline using a small-sample survey instrument known as the Knowledge, Practices, and Coverage (KPC) survey, based on DHS questions. Households were selected according to a standard cluster sampling methodology, with 30 independent clusters and 10 households in each cluster. Cluster selection was based on village level population data, with probability of selection proportional to population size. The method used is explained in detail elsewhere [9] The project target geographic area remained invariant from baseline to endline and comprised the entire district of Chokwe except Chokwe town – 48 villages with an estimated population of 119,467 at baseline). The KPC survey collects data on multiple indicators important to the project, and the sample is designed to detect statistically significant baseline/endline differences of at least ± 16% (alpha = 0.05, beta = 0.20) if no sub-sampling is done and an indicator starts at a baseline of 50%. Ninety five percent confidence intervals for project data used for LiST modelling are shown in Table 2. KPC surveys were carried out in October 1999 and July 2003. Project activities started in March 2000, so this is taken as the baseline year for LiST modelling.

KPC surveys cover mothers/caretakers of children 0-23 months of age. The surveys were carried out by the project staff themselves. In order to minimize possible bias, interviewers were not assigned clusters in which they themselves were working in their day-to-day project activities. The data was checked for consistency by an independent team at ICF Macro before being entered in a publically available database (http://www.mchipngo.net).

The other child health indicators in LiST for which the project did not have data were reviewed. Coverage data were estimated for most of the interventions being implemented at the time. The values of and sources for non-project data are shown in Table 3. Most data are from the 1997 and 2003 Demographic and Health Surveys (DHS). When 1997 data is used, its value assumed by LiST to change linearly toward its 2003 value, and the 2000 value assigned by LiST is used as the baseline to estimate mortality reductions.

**Table 3:** LiST indicators not in project data – estimated values and data sources

LiST Indicator	1997	2003	Data source
Antenatal Care	37.3%	52.3%	DHS, national data, 4 or more ANC visits
Folic acid supplementation or fortification	37.3%	52.3%	LiST calculates based on 4 or more ANC visits
Case management during pregnancy	1.9%	2.6%	LiST calculates as subcomponent of ANC
Syphilis detection and treatment	7.5%	26.1%	LiST calculates as subcomponent of ANC
Intermittent preventive treatment for malaria	0.0%	0.0%	No data available. Set at 0%
Tetanus toxoid vaccination x 2, last pregnancy	52.3%	68.8%	DHS, Gaza Province
Facility based birth / Skilled Birth Attendance	12%	12%	Estimated from project data - residual percentage of women not delivering with trained TBAs at endline
Essential Newborn Care	6.0%	6.0%	LiST calculates these coverage data as proportion of facility-based birth coverage
Basic Emergency Obstetric and Newborn Care	3.6%	3.6%	LiST calculates as a proportion of facility-based birth coverage
Comprehensive Obstetric and Newborn Care	2.4%	2.4%	LiST calculcates as a proportion of facility-based birth coverage
Antibiotics for preterm premature rupture of membranes	0.0%	0.0%	LiST calculates from facility-based coverage data
Newborn Resuscitation - Facility/Home	0/31.0%	0/31.0%	LiST calculates from facility and clean home delivery coverage
Exclusive Breastfeeding	30.2%	30.0%	DHS, national data
Vitamin A Supplementation	46.0%	54.7%	DHS 2003, Gaza Province data; 1997 data from reference [17]
DPT3 vaccine	84.7%	90.4%	DHS, Gaza Province data
Polio vaccine	83.9%	88.0%	DHS, Gaza Province data
BCG vaccine	96.7%	97.1%	DHS, Gaza Province data
Case management of severe neonatal infection	9.6%	9.6%	LiST calculates from DHS facility-based birth coverage data
Use of water connection within 30 minutes of home	83.0%	83.0%	Pregnancy history survey (Edward, et. al.). Baseline value for 1999.
Antibiotics for dysentery	Not avail.	Not avail.	No data available. Set at 20% for both 1997 and 2003.
Vitamin A for measles treatment	Not avail.	Not avail.	No data available. Set at 90% for both 1997 and 2003.

The change between 1997 and 2003 values is assumed to be linear, and LiST calculates the mortality impact based on the values calculated for the year 2000 which was used as the baseline for project indicators.

LiST interventions not being implemented to a significant extent in Mozambique at the time (coverage set to zero at baseline and final): child ART, PMTCT, preventive postnatal care, kangaroo mother care, active early detection of maternal and neonatal complications, multiple micronutrient supplementation, oral antibiotic case management of severe neonatal infections, injectable antibiotic case management of severe infections in neonates, zinc for prevention/treatment of diarrhea, rotavirus/Hib/pneumococcal vaccines.

### LiST modeling

LiST is a cohort model of child survival from 0-59 months of age. Its structure and assumption are described in detail elsewhere.[7,10] LiST provides estimates of the cause-specific child mortality impact of over 40 interventions with strong evidence of effect on child survival. The user must supply the values of changes in coverage for these interventions. LiST has country-specific baseline under-five and infant mortality rates and cause of death profiles needed for its calculations. These parameters can be manipulated by the user if desired. The Child Health Epidemiology Reference Group (CHERG) meets periodically to weigh published evidence, determine which interventions to include in the model and what effect sizes to assign them.[11] The under-five mortality modeling is contained within the Spectrum platform which models demographic trends, given assumptions about population growth rates and prevalence of use of family planning methods. [12] Version 4.2 of the LiST tool was used for modeling and was downloaded from the Johns Hopkins Institute for International Programs web site. [7] The under-five and infant mortality rates used were those specific to the project area at baseline, as measured in the pregnancy history and described in detail elsewhere. [8] National cause of death profiles, population structure, and fertility data were used.

All available coverage data both from the World Relief Mozambique project and other sources were examined to determine which coverage indicators matched those in LiST. The authors discussed the indicator definitions and corresponding coverage data that best fit the interventions in LiST. Eight project indicators (Table 2) were mapped to LiST interventions. The fit between project indicators and LiST was exact for seven of the indicators. For one LiST indicator (complementary feeding) the project had no direct data, but had intervened for a package of behavior change practices that included both maternal nutritional practices during pregnancy and child feeding practices. The project had data on the coverage for increased food intake during last pregnancy, and this was used as a proxy for child complementary feeding practices. Of the other 21 indicators in LiST for interventions being implemented in Mozambique at the time, information was available from other sources for eight; LiST estimates the value of nine others from available data (e.g. LiST estimates coverage for syphilis screening from ANC coverage). Non-project data used for LiST modelling is summarized in Table 3. In summary, data was available for all but four of the indicators in LiST for interventions being implemented in Mozambique in the relevant time period.

Sensitivity analyses were run for the LiST estimates, by varying all the parameters used in the model: Coverage data was varied within the limits of the 95% confidence intervals. The values assigned for intervention effectiveness, baseline mortality figures, and cause of death profiles were varied by ± 10% as well.

### Pregnancy history survey

Infant and child mortality were estimated for the period from two years before the start of project activities through one year after it ended (1998-2004), with calculations for these parameters at one year intervals. The information for these calculations was derived from a complete pregnancy history survey of 998 women in the project area, performed in May 2004. The questionnaire used was adapted from the birth history in the DHS 2003 women’s questionnaire and was implemented by a group of surveyors that included personnel from the National Institute of Statistics who had implemented the 2003 DHS, as described in detail in Edward, et.al. [8] The pregnancy history covered all pregnancies, but the period analyzed and reported was the period from March 1998 to February 2004. The baseline period used to match LiST estimates was March 2000 – February 2001 and the endline period used was March 2003-Feb. 2004.

An unpublished Fortran program written by one of the authors of Edward, et. al. takes as input the time period (beginning and end dates in months) and age group (minimum and maximum) for mortality estimation and calculates m(x) for this age group in the time period. Using the formula of Chiang [13] and the calculation of mean time lived in the interval for those dying in the interval, _1_q_0_ and _5_q_0_ were calculated and the data plotted in a lexis diagram. Standard errors were calculated assuming a Poisson distribution.

## Results

LiST modeled mortality estimates and the corresponding directly measured mortality estimates are shown in Table 4. The project had several measures of under-five and infant mortality reduction, derived both from a project-implemented community-based vital events registration system and from the independent pregnancy history survey. [8] The latter was felt to be the most accurate mortality measure for use as a comparison to LiST results. The directly measured under-five mortality rate (U5MR) from the birth history survey demonstrated a baseline value of 180 per 1,000 live births (95% CI, 130 – 230) in the 12 month period from March 2000 to February 2001 decreasing to an endline value of 114 per 1,000 live births (95% CI, 75 – 153) for the period from March 2003 to February 2004. This represents a 37% reduction in U5MR. The estimation of under-five mortality based on coverage changes modeled in LiST was 110, an estimated U5MR reduction of 39%. The directly measured infant mortality rate (IMR) from the birth history survey demonstrated a baseline value of 102 per 1,000 live births (95% CI, 64 – 141) for the period from March 2000 to February 2001 decreasing to an endline value of 53 per 1,000 live births (95% CI, 25 – 81) for the period from March 2003 to February 2004. This represents a 48% reduction in IMR. The estimation of IMR based on coverage changes modeled in LiST gave an endline IMR of 67, for an estimated IMR reduction of 34%. The LiST estimates for both under-five and infant mortality reductions are within the 95% confidence limits of the directly measured mortality estimates obtained from the pregnancy history survey.

**Table 4:** Under-five and infant mortality, comparison of measured and LiST modelled changes

Parameter	Baseline measured value (95% CI)	Endline measured value (95% CI)	Measured mortality reduction (%)	Endline modeled value	Absolute difference	Relative difference
**U5MR**	180 (130 – 230)	114 (75 – 153)	37%	110	4	4%
**IMR**	102 (64 – 141)	53 (25 – 81)	48%	67	14	26%

## Discussion

### Accuracy and completeness of coverage data

We used survey data generated as part of standard program monitoring and evaluation activities to model mortality impacts using LiST. Although the available data was not collected as part of a research project, the coverage data input into LiST was of sufficient quality to generate relatively accurate estimates within the limits of the tool. A standard survey instrument was used; data was collected by professional project staff; the potential for bias reduced by avoiding having interviewers collect information from villages where they worked; supervisory spot checks were performed for reliability of information; and data was reviewed for quality by technical support staff from ICF Macro on entry into the online child survival project database.

Although project interventions targeted children 0-59 month olds, which is the cohort modeled in LiST and whose mortality was measured directly in the pregnancy history, the coverage data used for LiST was collected for children 0-23 months old. The inaccuracy caused by this is likely to be small for the following reasons: (1) Even though the KPC measures are collected for children 0-23 months of age, in fact the project implemented interventions for the entire 0-59 month cohort of children, so we expect that the coverage for 0-59 month olds to be substantially the same. (2) we expect that 79% of deaths in children 0-59 months occurred in 0 to 23 month olds, so coverage of 0-23 month olds is the most critical. This calculation was done using the Model Life Tables for Developing Countries published by the United Nations [14] for an area with the project’s baseline U5MR and IMR. (3) LiST assigns the same effect size to relevant age groups 0-23 months and 24-59 months for all modelled project interventions. As part of the sensitivity analysis presented in Table 5, the effect was examined of halving the coverage change among 24-59 month olds compared to that measured in the KPC for 0-23 month olds. This dropped the estimate of U5MR reduction by 4.8%.

This project had several nutrition education interventions for well, sick, and malnourished children as well as pregnant mothers, but only an estimate for complementary feeding matched the nutrition interventions in LiST. The CHERG has not included other project interventions in LiST like continued feeding during diarrheal episodes because of a lack of published high quality data needed to accurately estimate an effect size, even though they are likely to have an effect on child mortality.

**Table 5:** Sensitivity analysis results for LiST modelling

Change in parameter modeled in LiST	Change in estimated decline in U5MR	Change in estimated decline in IMR
ITN coverage change raised 10%	Increase < 0.1%	Increase < 0.1%
ITN coverage change lowered 10%	Decrease < 0.1%	Decrease < 0.1%
ITN effect size raised 10%	Increase < 0.1%	Increase < 0.1%
ITN effect size lowered 10%	Decrease 0.6%	Decrease 1.0%
Baseline U5MR raised 10%	Increase 1.1%	Increase 0.6%
Baseline U5MR lowered 10%	Decrease 1.9%	Increase 0.6%
Proportion of diarrhea deaths raised 10%	Increase 1.3%	Increase 1.0%
Proportion of diarrhea deaths lowered 10%	Decrease 1.9%	Decrease 1.9%
All age-specific coverage changes among 24-59 month olds reduced to half that measured in the KPC surveys for 0-23 month olds	Decrease 4.8%	N/A

### Accuracy of modelled mortality estimates

The accuracy of the U5MR reduction estimate (39% LiST; 37% Pregnancy History) was better than the IMR reduction estimate (34% LiST; 48% Pregnancy History). Both were within the 95% CI of the parameter, but LiST’s underestimation of the reduction in IMR may be caused by the fact that the only nutritional intervention with probable infant mortality impact that could be modelled in LiST was complementary feeding.

The results of a sensitivity analysis of the LiST model are shown in Table 5. LiST estimates of mortality reduction are calculated based on several inputs: The baseline mortality rate, cause of death profile, change in coverage for each of the interventions in the model, and their effect sizes. Table 5 shows the effect on LiST’s estimates of the reductions in U5MR and IMR caused by changing one of the most critical examples of each of these parameters by 10%. The manipulations of the diarrhea and malaria parameters are shown in the table, as the project had large coverage changes for highly effective interventions for these causes of death. The modelled changes in mortality are more sensitive to changes in parameters that affect the calculation of the overall baseline mortality than they are to changes in the estimation of coverage or intervention effectiveness. This is not surprising, as the value of the baseline mortality affects the calculations for all interventions in the model.

One of the potential strengths of LiST is its ability to simplify analysis of situations in which multiple interventions are implemented simultaneously. Yet to date there have been few published reports on the accuracy of LiST estimates for mortality reductions in areas where packages of community-based child health interventions are being implemented. The LiST validation with data from the evaluation of Accelerated Child Survival and Development programs is similar to this one [15] and to some extent the national level exercises with DHS data.[16] The current analysis shows that even in the context of relatively complex community-based NGO programming with interventions designed to affect less proximate determinants of child health like level of community organization and women’s empowerment, LiST accurately estimated mortality changes.

### Limitations of validation analysis

Although coverage data for project interventions was fairly complete and the time periods coincided well with the mortality estimates calculated from the pregnancy history, the main limitations of the current exercise are (1) that the data was not available from the project for 21 relevant LiST indicators, and had to be estimated mainly from consecutive DHS surveys and (2) the 95% confidence intervals are quite wide for the mortality estimates derived from the pregnancy history.

There are cautions that must be kept in mind when using LiST. The accuracy of its estimates is dependent on having accurate information on the causes of death in the program area. National cause of death profiles are now available through CHERG, but there may be important variation from one region of a country to another. The outputs from LiST must also be interpreted in light of complementary considerations. For example, when used in planning LiST could mistakenly give the impression that mature interventions like vaccination that already have achieved high levels of coverage are not important, as simply maintaining high coverage yields no additional lives saved. LiST also does not take account of the mode of delivery and the fact that delivery of some interventions like antenatal care or vaccination establishes a platform that can serve for adding other interventions, like ITN or vitamin A distribution. Even with an awareness of these limitations and caveats, LiST can be a valuable aid in prioritizing choices for deployment of scarce resources.

Although only a single project was identified for study, it is typical of integrated, community-based NGO programming and implemented under realistic field conditions in a resource-constrained setting typical of conditions of other community-based NGO programming and the type of settings in which greater progress needs to be made to reach MDG4 targets.

## Conclusions

A validation exercise has confirmed that in a relatively routine field setting of an NGO child survival project implementing a package of community-based interventions in Mozambique, the Lives Saved Tool (LiST) provides a reasonably accurate estimate of under-five and infant mortality reduction when compared to independent directly measured mortality estimates. These are the kinds of routine programming conditions that LiST attempts to simulate with its modeling. These findings support the use of LiST as a practical tool for estimating the mortality effect of NGO community-based child health programs that is less costly than direct mortality measurement. These findings also support the use of LiST as a planning tool for choosing among child survival interventions in an attempt to maximize mortality impact in pursuit of MDG4.

## Competing interests

No financial or non-financial competing interests declared.

## Authors' contributions

JR did primary writing of the manuscript; DP performed LiST modelling exercises; HP, AE, MM, and PE contributed to the analysis of the validation study and writing of the manuscript; LR contributed to writing of the manuscript.

## Role of the funding source

Several authors (JR, DP, LR) have been associated with USAID’s Child Survival and Health Grants Program during manuscript preparation. Several others (MM, PE) were associated with World Relief, whose Vurhonga II project was funded by USAID through this mechanism. The study sponsors had no role in the study, data collection, or analysis. The corresponding author had final decision-making authority over interpretation of the results and the decision to submit this paper.

## References

[B1] Rajaratnam J Marcus J Flaxman A Neonatal, postneonatal, childhood, and under-5 mortality for 187 countries, 1970–2010: a systematic analysis of progress towards Millennium Development Goal 4 Lancet 2010 375 1988 2008 10.1016/S0140-6736(10)60703-9 20546887

[B2] Freeman P Perry H Gupta S Rassekh B Accelerating progress in achieving the millennium development goal for children through community-based approaches Global Public Health 2009 1 20 10.1080/17441690903330305 19890758

[B3] Bhutta Z Lassi Z Empowering communities for maternal and newborn health Lancet 2010 375 1142 1144 10.1016/S0140-6736(10)60324-8 20362800

[B4] Bhutta Z Samana A Cousens S Alma-Ata: Rebirth and Revision 6: Interventions to address maternal, newborn, and child survival: what difference can integrated primary health care strategies make? Lancet 2008 372 972 89 10.1016/S0140-6736(08)61407-5 18790320

[B5] Rosata M Laverack G Howard-Grabman L Community participation: lessons learned for maternal, newborn, and child health Lancet 2008 372 962 71 10.1016/S0140-6736(08)61406-3 18790319

[B6] Friberg I Bhutta Z Darmstadt G Comparing modelled predictions of neonatal mortality impacts using LiST with observed results of community-based intervention trials in South Asia Int J Epidemiol 2010 39 Suppl 1 i11 i20 10.1093/ije/dyq017 20348113 PMC2845856

[B7] Johns Hopkins Bloomberg School of Public Health Institute for International Programs, LiST home http://www.jhsph.edu/dept/ih/IIP/list/index.html

[B8] Edward A Ernst P Taylor C Examining the evidence of under-five mortality reduction in a community-based programme in Gaza, Mozambique Trans R Soc Trop Med Hyg 2007 101 8 814 822 10.1016/j.trstmh.2007.02.025 17482222

[B9] Maternal and Child Health Integrated Program PVO/NGO Support Knowledge, Practice and Coverage survey 2000 [Online] Available at: http://www.mchipngo.net/controllers/link.cfc?method=tools_modules_kpc2009 [Accessed October 2007, October 2009]

[B10] Victora C LiST: using epidemiology to guide child survival policymaking and programming Int 2010 39 suppl 1 i1 i2 10.1093/ije/dyq044 20348111 PMC2845876

[B11] Walker N Fischer-Walker C Bryce J Standards for CHERG reviews of intervention effects on child survival Int 2010 39 suppl 1 i21 i31 10.1093/ije/dyq036 20348122 PMC2845875

[B12] Stover J McKinnon R Winfrey B Spectrum: a model platform for linking maternal and child survival interventions with AIDS, family planning and demographic projections Int J Epidemiol 2010 39 suppl 1 i7 i10 10.1093/ije/dyq016 20348129 PMC2845855

[B13] Chiang C The Life Table and Its Applications 1984 Malabar, Florida: Robert E. Krieger Publ. Co.

[B14] United Nations Model Life Tables for Developing Countries 1982 Geneva Annex II. Accessed at http://www.un.org/esa/population/publications/Model_Life_Tables/Model_Life_Tables.htm

[B15] Hazel E Gilroy K Friberg I Comparing modelled to measured mortality reductions: applying the Lives Saved Tool to evaluation data from the Accelerated Child Survival Programme in West Africa Int J Epidemiol 2010 39 suppl 1 i32 i39 10.1093/ije/dyq019 20348124 PMC2845858

[B16] Masanja H de Savigny D Smithson P Child survival gains in Tanzania: analysis of data from demographic and health surveys Lancet 2008 371 1276 83 10.1016/S0140-6736(08)60562-0 18406862

[B17] Aguayo V Kahn S Ismael C Meershoek S Vitamin A deficiency and child mortality in Mozambique Public Health Nutrition 2005 8 1 29 31 10.1079/PHN2005664 15705242

